# Comparison of the CES-D and PHQ-9 depression scales in people with type 2 diabetes in Tehran, Iran

**DOI:** 10.1186/1471-244X-11-61

**Published:** 2011-04-16

**Authors:** Mohammad E Khamseh, Hamid R Baradaran, Anna Javanbakht, Maryam Mirghorbani, Zahra Yadollahi, Mojtaba Malek

**Affiliations:** 1 Endocrine Research Center (Firoozgar), Institute of Endocrinology and Metabolism, Tehran University of Medical Sciences, Iran; 2 Tehran Psychiatry Institute, Tehran University of Medical Sciences, Iran

## Abstract

**Background:**

The quality of life in patients with various chronic disorders, including diabetes has been directly affected by depression. Depression makes patients less likely to manage their self-care regimens. Accurate assessment of depression in diabetic populations is important to the treatment of depression in this group and may improve diabetes management. To our best knowledge, there are few studies that have looked for utilizing questionnaires in screening for depression among patients with diabetes in Iran. Therefore the aim of this study was to assess the efficacy and accuracy of the Center for Epidemiological Studies Depression (CES-D) scale and the Patient Health Questionnaire-9 (PHQ-9), in comparison with clinical interview in people with type 2 diabetes.

**Methods:**

Outpatients who attended diabetes clinics at IEM were recruited on a consecutive basis between February 2009 and July 2009. Inclusion criteria included patients with type 2 diabetes who could fluently read and speak Persian, had no severe diabetes complications and no history of psychological disorders. The history of psychological disorders was ascertained through patients' medical files, taking history of any medications in this regard. The study design was explained to all patients and informed consent was obtained. Volunteer patients completed the Persian version of the questionnaires (CES-D and PHQ-9) and a psychiatrist interviewed them based on Structured Clinical Interview (SCID) for DSM-IV criteria.

**Results:**

Of the 185 patients, 43.2% were diagnosed as having Major Depressive Disorder (MDD) based on the clinical interview, 47.6% with PHQ-9 and 61.62% with CES-D. The Area Under the Curve (AUC) for the total score of PHQ-9 was 0.829 ± 0.30. A cut-off score for PHQ-9 of ≥ 13 provided an optimal balance between sensitivity (73.80%) and specificity (76.20%). For CES-D the AUC for the total score was 0.861 ± 0.029. Optimal balance between sensitivity (78.80%) and specificity (77.1%) was provided at cut-off score of ≥ 23.

**Conclusions:**

It could be concluded that the PHQ-9 and CES-D perform well as screening instruments, but in diagnosing major depressive disorder, a formal diagnostic process following the PHQ-9 and also the CES-D remains essential.

## Background

The quality of life in patients with various chronic disorders, including diabetes has been directly affected by depression [1,2]. Depression makes patients less likely to manage their self-care regimens [3,4]. Based on a recent systematic review, the prevalence of depression was significantly higher in patients with Type 2 diabetes and it has been shown that people with diabetes are more likely to have higher rate of depression compared to their non diabetic counterparts [5].

Co-morbidity of depression and diabetes results in higher HbA1c levels [6,7], increased number and severity of complications and higher mortality rate [8-10]. Moreover, depression in patients with diabetes is associated with increased rate of medical symptoms reporting and health care seeking [10,11] more hospitalizations and hospitalization days [12] and higher healthcare costs [13,14] impaired patient-provider communication [15] and lower patient satisfaction [16] are other adverse consequences.

Therefore accurate assessment of depression in diabetic populations is important to the treatment of depression in this group and may improve diabetes management.

The gold standard for assessment of clinical depression could be a standardized, structured patient interview that yields clinical diagnoses that conform to Diagnostic and Statistical Manual of Psychiatric Disorders, 4th edition (DSM-IV) criteria. While time and cost restrict use of this method for screening purpose, self-administered questionnaires are easy to use and cost- effective. Several questionnaires have been developed such as Beck Depression Inventory [17], the Center for Epidemiological Studies Depression (CESD) scale [18], the Patient Health Questionnaire-9 [19] and the Center for Epidemiologic Studies Depression Scale Revised (CESD-R) which was recently created [20].

To our best knowledge, there are few studies that have looked for utilizing questionnaires in screening for depression among patients with diabetes in Iran. Therefore the aim of this study was to assess the efficacy and accuracy of these tools, (CESD) and (PHQ-9), in comparison with clinical interview in Iranian people with diabetes.

## Methods

This cross-sectional study was conducted at Institute of Endocrinology and Metabolism (IEM) affiliated to Tehran University of Medical Sciences, Tehran, Iran. Ethics approval was granted from the Ethics' Board at IEM. Outpatients who attended diabetes clinics at IEM were recruited on a consecutive basis between February 2009 and July 2009. Inclusion criteria included patients with type 2 diabetes who could fluently read and speak Persian, had no severe diabetes complications and no history of psychological disorders. The history of psychological disorders was ascertained through patients' medical files, taking history of any medications in this regard. The study design was explained to all patients and informed consent was obtained.

We employed two standard questionnaires, CES-D and PHQ-9, for this study. The PHQ-9 focuses on the nine signs and symptoms of depression from DSM-IV. The PHQ-9 offers a categorical algorithm for the diagnosis of depressive disorder. Major depression is diagnosed if 5 or more of the 9 depressive symptoms criteria have been present for at least "more than half the days" in the past 2 weeks (suicidal thoughts count if present at all) and one of the symptoms is depressed mood or anhedonia. In addition, the sum score (0-27) is used for screening purposes and for measuring depression severity. The cut-off point that is most widely used to indicate a positive case for depressive disorder is the sum score of 10 or higher [21]. CES-D is a 20-item questionnaire that assesses depressive symptoms over the previous 7 days. We used Cut-off points of 16 and 22 to define "likely depression" [18,21].

Using a standard 'forward-backward' translation procedure, the English language version of the questionnaires (CES-D and PHQ-9) were translated into Persian (Farsi). Then these questionnaires were piloted on 46 patients. The reliability of these questionnaires was measured by using Cronbach's alpha (CES-D-Cronbach's Alpha = 0.92 and PHQ-9-Cronbach's Alpha = 0.86).

The aims and details of the study were explained to patients when attending clinic by a trained nurse. Volunteer patients completed both questionnaires. Then scheduled appointments were made with a psychiatrist who was associate clinical professor of Tehran Psychiatry Institute (TPI), in the same week as completing the questionnaires. The psychiatrist was blind to results of these questionnaires and she interviewed patients based on Structured Clinical Interview (SCID) for DSM-IV (Persian Translation and Cultural Adaptation) [22]. The average duration of interview took between 20-40 minutes. The interview had implications only for research proposal however after diagnosis of depression for each patients, the psychiatrist started the necessary treatment and/or any medications for them. In addition demographic and clinical information were gathered at the time of administrating the questionnaires by that trained nurse.

### Statistical analysis

To determine the screening performance of the two questionnaires in identifying patients with MDD and to identify optimal cut-off scores, receiver operating characteristic curve (ROC) analysis was used. The Area Under the Curve (AUC) was calculated to quantify screening ability. The AUC of the screening instrument is evaluated by comparison with the AUC of the diagonal line, which represents classification by chance (AUC = 0.50). The optimal cut-off score of the screening instrument is selected by using the score that is closest to the intersection of the ROC and the diagonal line from the upper left to the lower right side of the graph. Descriptive data are given as mean ± SD and percentage. Comparison among subjects of groups was performed by student's t-test for continuous variables as well as Chi- square test for frequency of dichotomous variables. SPSS v.16 was used for statistical analyses. A p < 0.05 was considered significant.

## Results

Totally one hundred and eighty five patients completed the questionnaires and were interviewed by a psychiatrist. Approximately fifty-two percent of the patients were female. The mean age was 56.1(9.6) years, the mean of duration of diabetes was 9.8(SD = 7.3) years, and average HbA1C was 8.1(SD = 1.92) (Table 1).

**Table 1:** Demographic characteristics of study sample who had screened for depression by PHQ-9 and CES-D and Clinical Interview

*characteristic*	*Total sample*	*Clinical interview*			*PHQ-9*			*CES-D (score ≥ = 16)*			*CES-D (score ≥ = 22)*		
	*n = 185 (%)*	*MDD* *n = 80*	*No MDD* *n = 105*	*P*	*MDD* *n = 88*	*No MDD* *n = 97*	*P*	*MDD* *n = 114*	*No MDD* *n = 71*	*P*	*MDD* *n = 90*	*No MDD* *n = 95*	*P*
*Gender*													
*Male*	*89(48.1)*	*33(41.2)*	*56(53.3)*	*P = 0.10*	*30(34.1)*	*59(60.8)*	*P < 0.001*	*42(36.8)*	*47(66.2)*	*P < 0.001*	*31( 34.4)*	*58(61.1)*	*P < 0.001*
*Female*	*96(51.9)*	*47(58.8)*	*49(46.7)*		*58(65.9)*	*38(39.2)*		*72(63.2)*	*24(33.8)*		*59(65.6)*	*37(38.9)*	
*Education*													
*< 8 grades*	*100(54.1)*	*43(53.8)*	*57(54.3)*	*P = 0.94*	*54(61.4)*	*46(47.4)*	*P = 0.05*	*66(57.9)*	*34(47.9)*	*P = 0.18*	*53(58.9)*	*47(49.5)*	*P = 0.19*
*≥ 8grades*	*85(45.9)*	*37(46.2)*	*48(45.7)*		*34(38.6) *	*51(52.6)*		*48(42.1)*	*37(52.1)*		*37(41.1)*	*48(50.5)*	
*Insurance *													
*Yes*	*168(92.3)*	*74(93.7)*	*94(91.3)*	*P = 0.54 *	*80(90.9)*	*88(93.6)*	*P = 0.49*	*104(92.9)*	*64(91.4)*	*P = 0.72*	*83(92.2)*	*85(92.4)*	*P = 0.96*
*No*	*14(7.7)*	*5(6.3)*	*9(8.7)*		*8(9.1)*	*6(6.4)*		*8(7.1)*	*6(8.6)*		*7(7.8)*	*7(7.6)*	
*Medication*													
*Oral*	*121(67.6)*	*50(64.9)*	*71(69.6)*	*P = 0.36*	*54(62.1)*	*67(72.8)*	*P = 0.30*	*74(66.1)*	*47(70.1)*	*P = 0.83*	*54(60.7)*	*67(74.4)*	*P = 0.14*
*Insulin*	*25(14)*	*14(18.2)*	*11(10.8)*		*14(16.1)*	*11(12.0)*		*16(14.3)*	*9(13.4)*		*15(16.9)*	*10(11.1)*	
*Oral & Insulin*	*34(18.4)*	*13(16.9)*	*20(19.6)*		*19(21.8)*	*14(15.2)*		*22(19.6)*	*11(16.4)*		*20(22.5)*	*13(14.4)*	
*Family income*													
*low*	*95(51.4)*	*43(53.8)*	*52(49.5)*	*P = 0.56*	*49(55.7)*	*46(47.4)*	*P = 0.26*	*63(55.3)*	*32(45.1)*	*P = 0.17*	*49(54.4)*	*46(48.4)*	*P = 0.41*
*middle-high*	*90(48.6)*	*37(46.2)*	*53(50.5)*		*39(44.3)*	*51(52.6)*		*51(44.7)*	*39(54.9)*		*41(45.6)*	*49(51.6)*	
*Age (mean ± SD)*	*56.17 ± 9.60*	*54.38 ± 9.16*	*57.53 ± 9.74*	*P = 0.02*	*54.88 ± 10.13*	*57.34 ± 8.98*	*P = 0.08*	*55.87 ± 10.31*	*56.65 ± 8.37*	*P = 0.59*	*55.14 ± 10.32*	*57.14 ± 8.81*	*P = 0.15*
*HbA1C*	*8.10 ± 1.92*	*8.14 ± 1.98*	*8.06 ± 1.89*	*P = 0.80*	*8.32 ± 2.01*	*7.91 ± 1.84*	*P = 0.18*	*8.25 ± 1.99*	*7.86 ±1.79*	*P = 0.21*	*8.26 ± 2.01*	*7.95 ± 1.84*	*P = 0.32*
*BMI*	*28.33 ± 4.72*	*28.52 ± 4.33*	*28.20 ± 5.00*	*P = 0.68*	*28.55 ± 4.56*	*28.16 ± 4.87*	*P = 0.60*	*28.60 ± 4.68*	*27.92 ± 4.80*	*P = 0.38*	*28.58 ± 4.35*	*28.12 ± 5.03*	*P = 0.55*
*Diabetes duration (year ± SD )*	*9.83 ± 7.38*	*11.02 ± 7.26*	*8.93 ± 7.37*	*P = 0.05*	*9.22 ± 6.93*	*10.38 ± 7.75*	*P = 0.29*	*9.91 ± 7.06*	*9.70 ± 7.91*	*P = 0.85*	*9.77 ± 6.87*	*9.88 ± 7.86*	*P = 0.91*

Of the 185 patients, eighty (43.2%) were diagnosed as having Major Depressive Disorder (MDD) based on the clinical interview. Comparing those with MDD and without MDD, the former found to be younger and this difference was statistically significant (P = 0.02). These two groups were not different in other variables (Table 1).

The PHQ-9 diagnosed 88 (47.6%) patients with MDD. Women with depression were more dominant (P < 0.001).

On the CES-D, patients with MDD were found to be 114 (61.62%) and 90 (48.64%) with cut-points of ≥ 16 and ≥ 22, respectively. By considering both of cut-points, MDD was identified more in female than in male and this difference was statistically significant (P < 0.001).

**Figure 1:**
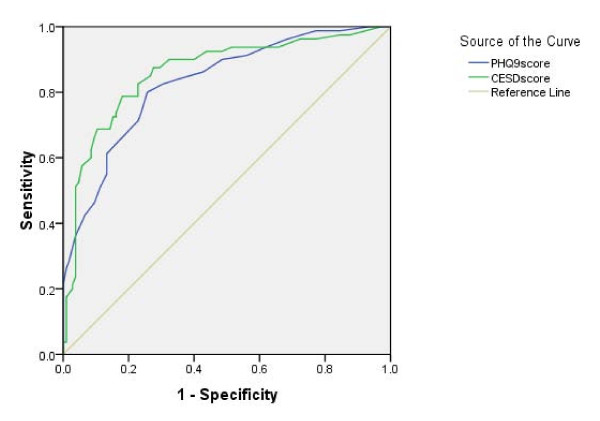
**Receiver operating curve analyses for major depression for each screening instrument**.

We compared the screening performance of each questionnaire with clinical interview (Table 2). The ability of the questionnaires to screen for MDD according to DSM-IV was assessed by using the area under the ROC (AUC) (Figure 1).

**Table 2:** Diagnostic performance of questionnaires for detection of major depressive disorder

	Sensitivity %	Specificity %	+ LR	- LR
PHQ-9 algorithm	77.5 (66.5-85.7)	75.2(65.6-82.9)	3.1(2.1-4.4)	0.2(0.1-0.4)
PHQ-9(score ≥ 10)	83.8(73.4-90.7)	65.7(55.7-74.5)	2.4(1.8-3.2)	0.2(0.1-0.4)
PHQ-9(score ≥ 13)	73.8(62.5-82.6)	76.2(66.6-83.7)	3.1(2.1-4.4)	0.3(0.2-0.5)
CES-D(score ≥ 16)	90 (80.7-95.2)	60 (49.9-69.2)	2.2(1.7-2.8)	0.2(0.0-0.3)
CES-D(score ≥ 22)	82.5(72.-89.7)	77.1(67.7-84.5)	3.6(2.5-5.2)	0.2(0.1-0.3)
CES-D(score ≥ 23)	78.8(67.8-86.7)	77.1(67.7-84.5)	3.4(2.3-4.9)	0.2(0.1-0.4)

The AUC for the total score of PHQ-9 was 0.829 ± 0.30, which is significantly higher than the diagonal line (P < 0.001). A cut-off score for PHQ-9 of ≥13 provided an optimal balance between sensitivity (73.80%) and specificity (76.20%). For CES-D the AUC for the total score was 0.861 ± 0.029 which is significantly higher (p < 0.001) than the diagonal line as well. Optimal balance between sensitivity (78.80%) and specificity (77.1%) was provided at cut-off score of ≥ 23.

The reliability of these questionnaires was measured by using Cronbach's alpha (CES-D Cronbach's alpha = 0.936 and PHQ-9 Cronbach's alpha = 0.873).

## Discussion

In this study, 43.2% of patients were diagnosed to have MDD by clinical interview. A recent systematic review estimated the prevalence of depression in adults with Type 2 diabetes compared to those without diabetes and the prevalence rate of depression was nearly twice as high in patients with diabetes compared to those without. (OR = 1.6, 95% CI = 1.5-1.7) [5]. In line with other studies, a report from Iran indicated that rate of depression in patients with diabetes was higher than those without diabetes (OR = 2.1, 95% CI 1.4-3.2) [23]. Other reports from Iran using different tools for depression showed high rates of depression in people with diabetes in Iranian population [24,25].

Anderson and colleagues stated that the prevalence of depression varied systematically as a function of the method used to identify depression cases and the study design. Furthermore, in both controlled and uncontrolled studies, depression rates were approximately two to three times higher in studies that used self-report measures versus diagnostic interview [26].

In our sample, rate of MDD was higher compared to previous findings [5] which could be explained by the fact that the specialized diabetes center may have attracted patients who had more problems, including more depression, than the non-referral patients with diabetes.

The main objectives of our study were to determine the accuracy of PHQ-9 and CES-D questionnaires in screening for major depressive disorder in Iranian patients with type 2 diabetes.

Sensitivity and specificity of the PHQ-9 in this study differ from previous accuracy studies [27,28] due to different prevalence of MDD in the populations. In our sample, applying algorithmic approach led to almost similar LRs as using scores. Considering these likelihood ratios, the PHQ-9 generates small to moderate shifts in pre- to posttest probability [29] of MDD in patients with diabetes indicating that the PHQ-9 might not be a proper tool to be used as a diagnostic instrument in a population at high risk of depression. It can be used in general practice for case finding, but should always be followed by diagnostic interview. Wittkampf and colleagues reported similar findings as our study [27].

Also the CES-D has different sensitivity and specificity compared to previous studies [21]. In our study, test characteristics of the CES-D are almost similar to the PHQ-9, indicating that the likelihood ratios alter posttest probability of MDD to a small to moderate degree. Therefore CES-D seems insufficient clinical tool for diagnosis of MDD in patients with diabetes.

Another important issue is that exclusion criteria in diagnosis of MDD are not included in the questionnaires so further assessment by clinical interview seems to be reasonable.

In this study, the PHQ-9 had AUC = 0.829 ± 0.30 and the CES-D had the AUC = 0.861 ± 0.029. However this difference was not statistically significant (P = 0.153). Therefore it seems no preference of employing one of these questionnaires.

Based on our experience from this study the depression symptoms of patients could be demonstrated easily and better by items of the CES-D. However, the PHQ-9 includes fewer items and it would be less time consuming to complete it.

The finding of this study has demonstrated that these questionnaires are valid and reliable in Persian language therefore they can be employed in Iranian population.

## Conclusions

It could be concluded that the PHQ-9 and CES-D (Farsi/Persian versions) perform well as screening instruments, but in diagnosing major depressive disorder, a formal diagnostic process following the PHQ-9 and also CES-D remains essential.

## Competing interests

The authors declare that they have no competing interests.

## Authors' contributions

All authors were involved in the conceptualisation of the study idea, development of the study design and preparation of the final manuscript. AJ, MM, MEK, HRB and ZY were also involved in the development of instruments, supervision of data collection and analysis. ZY is a consultant psychiatrist who carried out clinical interview with patients. All authors contributed to and approved the final manuscript

## Pre-publication history

The pre-publication history for this paper can be accessed here:

http://www.biomedcentral.com/1471-244X/11/61/prepub

## References

[B1] Schram MT Baan CA Pouwer F Depression and Quality of Life in Patients with Diabetes: A Systematic Review from the European Depression in Diabetes (EDID) Research Consortium Current Diabetes Reviews 2009 5 112 119 10.2174/157339909788166828 19442096 PMC2764861

[B2] Moussavi S Chatterji S Verdes E Tandon A Patel V Ustun B Depression, chronic diseases, and decrements in health: results from the World Health Surveys Lancet 2007 370 851 858 10.1016/S0140-6736(07)61415-9 17826170

[B3] Egede LE Ellis C Grubaugh AL The effect of depression on self-care behaviors and quality of care in a national sample of adults with diabetes General Hospital Psychiatry 2009 31 422 427 10.1016/j.genhosppsych.2009.06.007 19703635

[B4] Gonzalez JS Safren SA Cagliero E Wexler DJ Delahanty L Wittenberg E Blais MA Meigs JB Grant RW Depression, self-care, and medication adherence in type 2 diabetes: relationships across the full range of symptom severity Diabetes Care 2007 30 2222 2227 10.2337/dc07-0158 17536067 PMC4440862

[B5] Ali S Stone MA Peters JL Davies MJ Khunti K The prevalence of co-morbid depression in adults with Type 2 diabetes: a systematic review and meta-analysis Diabet Med 2006 23 1165 1173 10.1111/j.1464-5491.2006.01943.x 17054590

[B6] Park H Hong Y Lee H Ha E Sung Y Individuals with type 2 diabetes and depressive symptoms exhibited lower adherence with self-care Clin Epidemiol 2004 57 978 984 10.1016/j.jclinepi.2004.01.015 15504641

[B7] Lustman PJ Anderson RJ Freedland KE de Groot M Carney RM Clouseet RE Depression and poor glycemic control: a meta-analytic review of the literature Diabetes Care 2000 23 934 942 10.2337/diacare.23.7.934 10895843

[B8] Lin E Heckbert SR Rutter CM Katon WJ Ciechanowski P Ludman EJ Oliver M Young BA McCulloch DK Von Korff M Depression and Increased Mortality in Diabetes: Unexpected Causes of Death Ann Fam Med 2009 7 414 421 10.1370/afm.998 19752469 PMC2746517

[B9] Katon WJ Rutter C Simon G Lin E Ludman EJ Ciechanowski P Kinder L Young B Von Korff M The association of comorbid depression with mortality in patients with type 2 diabetes Diabetes Care 2005 28 2668 2672 10.2337/diacare.28.11.2668 16249537

[B10] Ludman EJ Katon WJ Russo J von Korff M Simon G Ciechanowski P Lin E Bush T Walker E Young B Depression and diabetes symptom burden Gen Hosp Psychiatry 2004 26 430 436 10.1016/j.genhosppsych.2004.08.010 15567208

[B11] Ciechanowski PS Katon WJ Russo JE Hirsch B The relationship of depressive symptoms to symptom reporting, self-care and glucose control in diabetes Gen Hosp Psychiatry 2003 25 246 252 10.1016/S0163-8343(03)00055-0 12850656

[B12] Subramaniam M Sum CF Pek E Stahl D Verma S Liow HP Chua CH Abdin E Chong AS Comorbid depression and increased health care utilization in individuals with diabetes General Hospital Psychiatry 2009 31 220 224 10.1016/j.genhosppsych.2009.01.001 19410100

[B13] Simon GE Katon WJ Lin EH Ludman E VonKorff M Ciechanowski P Young BA Diabetes complications and depression as predictors of health service costs Gen Hosp Psychiatry 2005 27 344 351 10.1016/j.genhosppsych.2005.04.008 16168795

[B14] Egede LE Zheng D Simpson K Comorbid depression is associated with increased health care use and expenditures in individuals with diabetes Diabetes Care 2002 25 464 470 10.2337/diacare.25.3.464 11874931

[B15] Piette JD Schillinger D Potter MB Heisler M Dimensions of patient-provider communication and diabetes self-care in an ethnically-diverse population J Gen Intern Med 2003 18 1 10 10.1046/j.1525-1497.2003.31968.x 12911644 PMC1494904

[B16] Katon WJ Clinical and health services relationships between major depression, depressive symptoms, and general medical illness Biol Psychiatry 2003 54 216 226 10.1016/S0006-3223(03)00273-7 12893098

[B17] Beck AT Ward CH Mendelson M Mock J Erbaugh J An inventory for measuring depression Arch Gen Psychiatry 1961 4 561 571 13688369 10.1001/archpsyc.1961.01710120031004

[B18] Radloff LS The CES-D scale: a self-report depression scale for research in the general population Applied Psychol Meas 1977 3 385 401 10.1177/014662167700100306

[B19] Kroenke K Spitzer RL Williams JB The PHQ-9: Validity of a brief depression severity measure Gen Intern Med 2001 16 606 613 10.1046/j.1525-1497.2001.016009606.x PMC1495268 11556941

[B20] Van Dam NT Earleywine M Validation of the Center for Epidemiologic Studies Depression Scale-Revised (CESD-R): Pragmatic depression assessment in the general population Psychiatry Res 2011 186 1 128 32 10.1016/j.psychres.2010.08.018 20843557

[B21] Hermanns N Kulzer B Krichbaum M Kubiak T Haak T How to screen for depression and emotional problems in patients with diabetes: comparison of screening characteristics of depression questionnaires, measurement of diabetes-specific emotional problems and standard clinical assessment Diabetologia 2006 49 469 477 10.1007/s00125-005-0094-2 16432706

[B22] Sharifi V Assadi SM Mohammadi MR Amini H Kaviani H Semnani Y Shabani A Shahrivar Z Davari-Ashtiani R Hakim Shooshtari M Seddigh A Jalali M Structured Clinical Interview for DSM-IV (SCID): Persian Translation and Cultural Adaptation Iran J Psychiatry 2007 1 46 48 10.1016/j.comppsych.2008.04.004 19059520

[B23] Khamseh ME Baradaran HR Rajabali H Depression and diabetes in Iranian patients: a comparative study Int J Psychiatry in Medicine 2007 37 81 86 10.2190/FP64-82V3-1741-842V 17645200

[B24] Larijani B Bayat MK Gorgani MK Bandarian F Akhondzadeh S Sadjadi SA Association between depression and diabetes German Journal of Psychiatry 2004 7 4 62 65

[B25] Safa AN Larijani B Shariati B Amini H Rezagholizadeh A Depression, quality of life and glycemic control in patients with diabetes Iranian Journal of Diabetes and Lipid Disorders 2008 7 2 195 204

[B26] Anderson RJ Freeland KE Clouse RE Lustman PJ The prevalence of comorbid depression in adults with diabetes: a meta-analysis Diabetes Care 2001 24 1069 78 10.2337/diacare.24.6.1069 11375373

[B27] Wittkampf K Ravesteijn H Baas K Hoogen H Schene A Bindels P Lucassen P van de Lisdonk E van Weert H The accuracy of Patient Health Questionnaire-9 in detecting depression and measuring depression severity in high-risk groups in primary care General Hospital Psychiatry 2009 31 451 459 10.1016/j.genhosppsych.2009.06.001 19703639

[B28] Wittkampf K van Ravesteijn H Baas K van de Hoogen H Schene A Bindels P Lucassen P van de Lisdonk E van Weert H Diagnostic accuracy of the mood module of the Patient Health Questionnaire: a systematic review General Hospital Psychiatry 2007 29 388 395 10.1016/j.genhosppsych.2007.06.004 17888804

[B29] Guyatt G Rennie D Users' guide to the medical literature 2002 8411578

